# Measuring governance at health facility level: developing and validation of simple governance tool in Zambia

**DOI:** 10.1186/1472-698X-13-34

**Published:** 2013-08-09

**Authors:** Wilbroad Mutale, Margaret Tembo Mwanamwenge, Dina Balabanova, Neil Spicer, Helen Ayles

**Affiliations:** 1 Department of community Medicine, University of Zambia School of Medicine, Lusaka, Zambia; 2 Clinical research Department, Faculty of Infectious and Tropical Diseases, London School of Hygiene and Tropical Medicine, London, UK; 3 ZAMBART Project, Ridgeway Campus, University of Zambia, Lusaka, Zambia; 4 Department of Global Health and Development, Faculty of Public Health and Policy, London School of Hygiene and Tropical Medicine, London, UK

## Abstract

**Background:**

Governance has been cited as a key determinant of economic growth, social advancement and overall development. Achievement of millennium development goals is partly dependant on governance practices. In 2007, Health Systems 20/20 conducted an Internet-based survey on the practice of good governance. The survey posed a set of good practices related to health governance and asked respondents to indicate whether their experience confirmed or disconfirmed those practices. We applied the 17 governance statements in rural health facilities of Zambia. The aim was to establish whether the statements were reliable and valid for assessing governance practices at primary care level.

**Methods:**

Both quantitative and qualitative methods were used. We first applied the governance statements developed by the health system 20/20 and then conducted focus group discussion and In-depth interviews to explore some elements of governance including accountability and community participation. The target respondents were the health facility management team and community members. The sample size include 42 health facilities. Data was analyzed using SPSS version 17 and Nvivo version 9.

**Results:**

The 95% one-sided confidence interval for Cronbach’s alpha was between 0.69 and 0.74 for the 16 items.

The mean score for most of the items was above 3. Factor analysis yielded five principle components: Transparency, community participation, Intelligence & vision, Accountability and Regulation & oversight. Most of the items (6) clustered around the transparency latent factor. Chongwe district performed poorly in overall mean governance score and across the five domains of governance. The overall scores in Chongwe ranged between 51 and 94% with the mean of 80%. Kafue and Luangwa districts had similar overall mean governance scores (88%). Community participation was generally low. Generally, it was noted that community members lacked capacity to hold health workers accountable for drugs and medical supplies.

**Conclusions:**

The study successfully validated and applied the new tool for evaluating health system governance at health facility level. The results have shown that it is feasible to measure governance practices at health facility level and that the adapted tool is fairly reliable with the 95% one-sided confidence interval for Cronbach’s alpha laying between 0.69 and 0.74 for the 16 items. Caution should be taken when interpreting overall scores as they tended to mask domain specific variations.

## Background

The word “Governance” is difficult to define. It‘s use may be associated with a set of principles, the exercise of legitimate authority through law and regulation, or processes for ensuring accountability and managing risk within organizations [1,2]. There are complex relationships within and across local, national and global levels of governance [2]. Governance has dimensions which must be considered when evaluating governance practices. The three dimensions commonly cited are political, economical and institutional. The political dimension refers to the process by which governments are selected, monitored and replaced. The economical dimension refers to the capacity of the government to effectively formulate and implement sound policies, including management of public resources. The institutional dimension includes the respect of citizens and the institutions that govern economic and social interactions among them [3].

Governance has been defined by the United Nations Development Programme (UNDP) as the exercise of political, economic and administrative authority in the management of a country’s affairs at all levels. Brinkerhoff et al., defined governance as the rules that distribute roles and responsibility among societal actors and that shape the interaction among them [4]. The United Kingdom Department for International Development (DFID) defined governance in terms of institutions, rules and systems of the state. The World Bank has taken the economical view of governance defining it as economic policy making and implementation with a focus on accountability and use of public resources [5]. Governance goes beyond government to include relationships and networks at various levels. It must be acknowledged that the concept of governance is not a coherent or agreed theoretical concept and there are debates about the nature of governance

Governance has been cited as a key determinant of economic growth, social advancement and overall development [6]. Research has shown that the modes of governance may influence health outcomes through their association with patterns of incentives and with regulatory and performance management regimes [7]. The achievement of millennium development goals is partly dependant on governance practices in low and middle income countries [6].

Health system governance concerns the actions and the means adopted by a society to organise itself in the promotion and protection of the health of the population. The rules defining such organization and its functioning, can be formal or informal [8].

Health systems contain three categories of actors: government, providers, and beneficiaries/clients. Health governance involves the rules that determine the roles and responsibilities of each of these categories of actors, and the relationships, structures, and procedures that connect them. Good governance in health reflects the application of a set of normative principles: accountability to patients and the broader public, an open policy process where competing interest groups operate on a level playing field, state capacity and legitimacy to manage the policy process and implement health policy decisions, effective and responsive service delivery, and the participation of civil society and private sector actors in both policymaking and service delivery [9].

In its health system building blocks which include service delivery, human resources, health information, Medical supplies, finance and governance, WHO has emphasised governance or stewardship as crucial in health system strengthening. WHO acknowledges that governance is one of the most complex building blocks. It involves overseeing and guiding the whole health system, private as well as public, in order to protect the public interest. This requires both political and technical action, because it involves reconciling competing demands for limited resources. With increasing demands for transparency and accountability the role of health system governance has become even more important [10].

Good governance should, in theory, lead to better performance. More accountability to beneficiaries can be an incentive for health officials and providers to improve services [11,12].

Thus to achieve a system of good health governance, a number of areas need to be addressed. These include improving the policy process through ensuring policy‒making based on evidence and open, informed, fair and equitable involvement of key stakeholders. Community participation has to be enhanced through increasing local information and leadership, and institutional incentives and openness of officials. Corruption has to be reduced, through tracking financial flows and disseminating information, auditing and citizen oversight [13].

Saddiqi et al., proposed 10 principles for assessing governance of the health system. These were strategic vision, participation and consensus orientation, rule of law, transparency, responsiveness, equity and inclusiveness, effectiveness and efficiency, accountability, intelligence and information and ethics [6].

In 2007, Health Systems 20/20 conducted an Internet-based survey on the practice of good governance in the health sector in collaboration with the Health Systems Action Network (HSAN). The survey posed a set of good practices related to health governance and asked respondents to indicate whether their experience confirmed or disconfirmed those practices. 17 questions were subsequently distilled from the semi structured and qualitative questions that represented statements about good health system governance. The responders were mainly mid level managers and the focus was at national level rather than primary care [9]. These questions were also used to assess governance practices in Rwanda as part of health system strengthening intervention [14]. However, these statements have not been validated for regular use in evaluating health system governance especially in rural settings were the concepts of governance may be less clear. We applied the 17 governance statements and adapted the statements to fit the primary care health workers working in rural health facilities of Zambia. The aim was to establish whether the statements were valid for assessing governance practices at primary care level and to identify the latent factors or domains of governance that were captured in the 17 statements or items. This was done as part of the baseline study.

### Methods

This work is part of larger study in Zambia known as Better Health through Mentoring and Assessment (BHOMA) which is a randomised step wedged community intervention that aims to strengthen the health system in three rural districts of Zambia. There are 42 target health facilities in the three study. The full methodology of the main study is described elsewhere [15] (*Personal communication*). In this study we used the governance tool developed by the health system 20/20 for measuring health system governance in the 42 health facilities. It contains 17 semi structured statements about good governance practices [9,14]. The answers were graded between 1 and 4 (4 = Agree 3 = Some what agree 2 = Some what disagree 1 = Disagree).The target respondents were the health facility management team in the rural health centres of Zambia. These were mainly the health facility incharge, clinical officers, nurses, environmental health technicians, pharmacists and in some places Classified Daily Employees (CDEs) who are usually lay workers working at health facility either voluntarily or are on government payroll. After explaining the self administered tool to the team they were then allowed to sit on their own and read each statement and then graded the performance of the health facility on each statement. They were only to come up with consensus responses on each statement. The teams consisted of 2–10 members with an average of 3 members per health facility. The research team did not take part in the grading and did not sit in the room where the grading was being done. The tool was pre-tested in pilot facilities which had settings similar to the study sites.

Principal factor analysis with Varimax and Kaiser Normalisation was used to determine the latent governance factors captured in the 17 statements. After factor analysis 16 statements had a coefficient above 0.4 and thus were retained for further analysis. Reliability test for the 16 items was done using cronbach’s alpha.

The maximum possible score by each health facility was 64.These scores were converted to percentage for easy comparisons. The total district score was calculated by the sum of individual health facility scores. After identification of the latent factors these were analyzed separately as domains which made the overall governance score.

Ethical approval was obtained from University of Zambia Biomedical Research Ethics Committee and London school of Hygiene and Tropical Medicine.

All participants signed written consent before taking part in the study. Confidentiality was maintained during data collection and publication.

### Qualitative data

For the qualitative component of the study, nine health facilities were selected from the three districts. The selection criterion was that in each district one rural, one semirural and one urban health facility was to be included. At each facility, In-depth interviews (IDI) were conducted with the health centre in-charge, Chairman of the Neighbourhood health committee (NHC) and a pharmacist were interviewed. Around the catchment area of each health facility, two Focus Group Discussions (FGDs) were held with men and women. In total 30 IDIs and 18 FGDs were conducted.

Qualitative data was analyzed using Nvivo version 9. The full methodology and results of the qualitative study are reported elsewhere [15]. Here we report on two elements of governance: Community participation and Accountability for medical supplies.

## Results

### Descriptive

#### Reliability of the 16 item scale

The 95% one-sided confidence interval for Cronbach’s alpha for the 16 item scale for governance was between 0.69 and 0.74.

The mean score for most of the items was above 3.The lowest mean was 2.48 which referred to “There being a mechanism for correcting those not complying with standards and code of conduct “followed by the mean of 2.86 for the “health facility having protocols for adult, child and maternal health services from the MoH(Table 1).

**Table 1:** Showing the mean scores across the 16 items for governance

	***N***	***Min***	***Max***	***Mean***	***Std.***
Systems exist for reporting, investigating, and adjudicating misallocation or misuse of resources.	42	1	4	3.19	1.131
The public have regular opportunities to meet with managers of the health facility to raise issues about service efficiency or quality.	42	1	4	3.60	.828
Local organisations and health service users have influence on what services are offered at the health facility.	42	1	4	3.31	.950
There are forums and procedures that give the public, technical experts, and local communities’ opportunities to provide input.	42	3	4	3.71	.457
The health facility use evidence on program results, patient satisfaction, and other health-related information to improve the services they deliver.	42	1	4	3.33	.979
Health facility managers rely on research data from health facility to plan services.	42	3	4	3.86	.354
The health facility regularly organize forums to solicit input from the public and concerned stakeholders.	42	1	4	3.36	.906
The health facility has protocols for adult, child and maternal health services from the MoH.	42	1	4	2.86	1.317
The facility managers ensure that Health workers follow protocols, standards and codes of conduct.	42	3	4	3.83	.377
The health facility collects and analyses local data.	42	2	4	3.81	.455
The health facilities receive regular external quality check team to ensure that the protocols and standards are followed.	42	1	4	3.52	.943
The allocation and utilization of resources are regularly tracked and information on results is available for review by the local communities/stakeholder.	42	1	4	3.43	.966
There is a mechanism for correcting those not complying with standards and code of conduct.	42	1	4	2.48	1.194
The public and concerned stakeholders have the capacity to advocate and participate effectively with the health facility officials in making plans.	42	1	4	3.19	.969
There are procedures and systems that clients, providers, and concerned stakeholders can use to fight bias and inequity in accessing health service.	42	1	4	3.38	.909
Health services are organised and financed in ways that offer incentives to health workers and community health workers to improve performance.	42	1	4	2.88	1.131

#### Factor analysis

Five latent factors were identified from factor analysis. Six (6) items loaded on the transparency latent factor, two (2) items loaded on the regulation & Oversight latent factor, three (3) items loaded on community participation. Three (3) items loaded on the intelligence & vision while two items loaded on accountability latent factor.

In the Transparency latent factors highest loading of 0.776 was in the item relating to “facilities receiving regular external quality check team to ensure that the protocols and standards are followed”. In the regulation & oversight factor the highest loading of 0.834 was in the item “managers ensure that Health workers follow protocols, standards and codes of conduct”. In community participation latent factor highest loading of 0.781 was in the item “Local organizations and health service users have influence on what services are offered at the health facility”. In the intelligence and vision latent factor, the highest loading of 0.827 was in the item “Health facility managers rely on research data from health facility to plan services”. In the accountability latent factors the highest loading of 0.783 was in the item “Systems exist for reporting, investigating, and adjudicating misallocation or misuse of resources” (Table 2).

**Table 2:** Factor analysis for the 16 item governance score

	**Latent factor**				
	**Transparency**	**Regulation & oversight**	**Community participation**	**Intelligence & Vision**	**accountability**
The health facilities receive regular external quality check team to ensure that the protocols and standards are followed.	**.776**				
The allocation and utilization of resources are regularly tracked and information on results is available for review by the local communities/stakeholder.	**.724**				
There is a mechanism for correcting those not complying with standards and code of conduct.	**.654**				
The public and concerned stakeholders have the capacity to advocate and participate effectively with the health facility officials in making plans.	**.617**		.		
There are procedures and systems that clients, providers, and concerned stakeholders can use to fight bias and inequity in accessing health service.	**.610**				
Health services are organised and financed in ways that offer incentives to health workers and community health workers to improve performance.	**.566**				
The facility managers ensure that Health workers follow protocols, standards and codes of conduct.		**.834**			
The health facility collects and analyses local data.		**.797**			
Local organisations and health service users have influence on what services are offered at the health facility.			**.781**		
There are forums and procedures that give the public, technical experts, and local communities’ opportunities to provide input.			**.768**		
The health facility use evidence on program results, patient satisfaction, and other health-related information to improve the services they deliver.			**.546**		
Health facility managers rely on research data from health facility to plan services.				**.827**	
The health facility regularly organize forums to solicit input from the public and concerned stakeholders.				**.685**	
The health facility has protocols for adult, child and maternal health services from the MoH.				**.501**	
Systems exist for reporting, investigating, and adjudicating misallocation or misuse of resources.					**.783**
The public/concerned stakeholders have regular opportunities to meet with managers of the health facility to raise issues about service efficiency or quality.					**.763**

*Extraction Method*: *Principal Component Analysis*. *Rotation Method*: *Varimax with Kaiser Normalization*.

### Sub group analysis of governance domains

#### District governance score

Chongwe performed poorly in overall mean governance score and across the five domains of governance. The overall scores in Chongwe ranged between 51 and 94% with the mean of 80%. For Chongwe district, the highest score by domain was in regulation and oversight (95%) and the lowest score was in Transparency domain (73%). Kafue and Luangwa had similar overall mean governance scores (88%). For Kafue the scores ranged between 75 and 98%. The highest score was noted in the regulation & oversight (95%) and the lowest scores in the transparency domain (83%). Luangwa district scores ranged between 73 and 98%. The highest score was in accountability (96%) lowest scores were in intelligence & vision domain.

**Figure 1:**
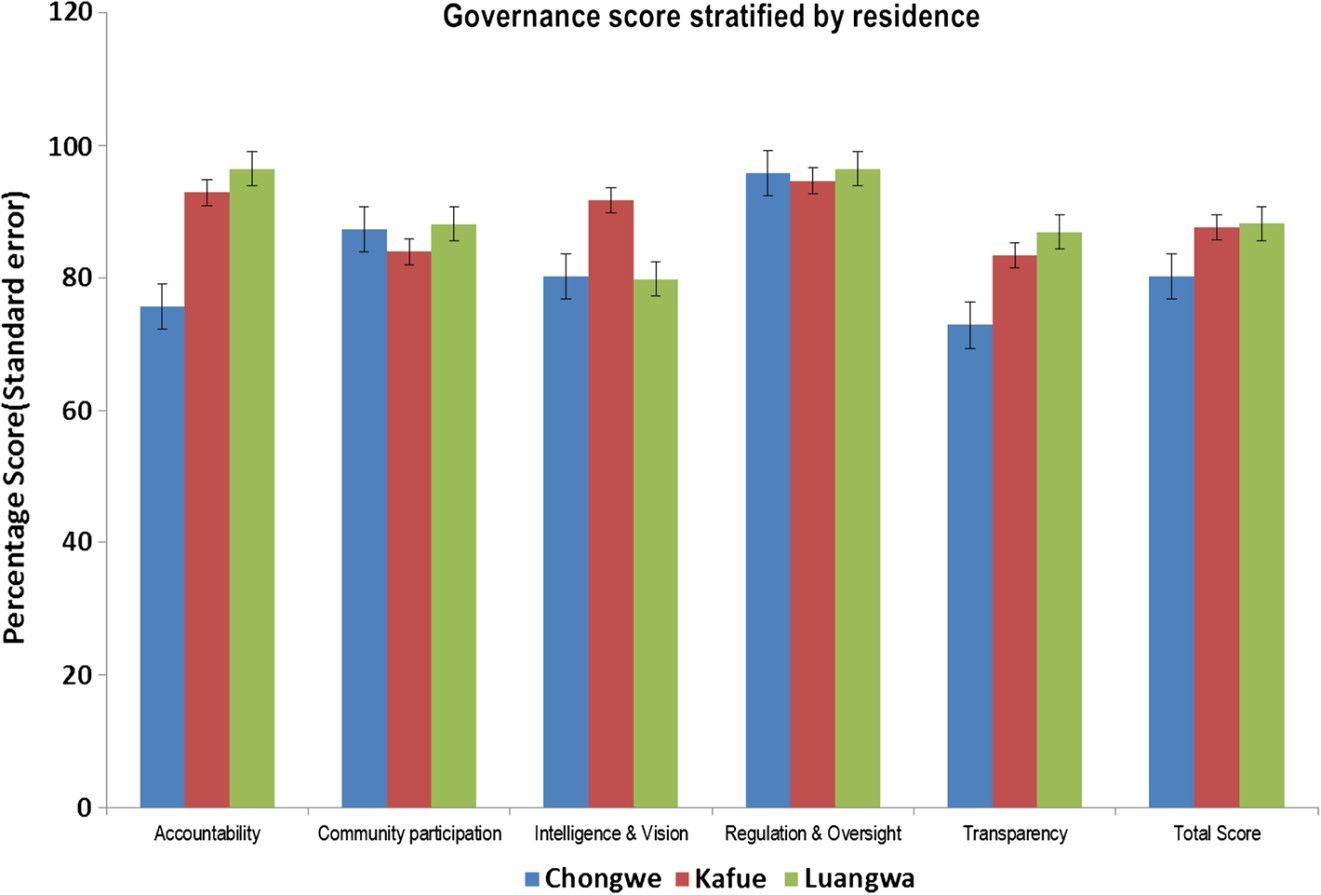
Governance scores stratified by district.

When domains were compared across the three districts, Accountability and transparency domains were highest in Luangwa and lowest in Chongwe. Intelligence & vision sub scores were highest in Kafue. Regulation & Oversight showed less variation across the districts (Table 3 and Figure 1).

**Table 3:** Governance score stratified by district

**District**	**Domain**	**N**	**Mini**	**Max**	**Mean**	**Std.**
**Chongwe**	Accountability	21	25.00	100.00	75.5952	25.76219
	Community participation	21	50.00	100.00	87.3016	17.79885
	Intelligence & Vision	21	41.67	100.00	80.1587	17.77096
	Regulation & Oversight	21	75.00	100.00	95.8333	8.22851
	Transparency	21	25.00	100.00	72.8175	20.81745
	**Total Score**	**21**	**51.56**	**93.75**	**80.1339**	**11.47287**
**Kafue**	Accountability	14	75.00	100.00	92.8571	8.07758
	Community participation	14	50.00	100.00	83.9286	14.42053
	Intelligence & Vision	14	58.33	100.00	91.6667	12.65924
	Regulation & Oversight	14	62.50	100.00	94.6429	11.72018
	Transparency	14	66.67	95.83	83.3333	9.80581
	**Total Score**	**14**	**75.00**	**98.44**	**87.6116**	**7.18552**
**Luangwa**	Accountability	7	75.00	100.00	96.4286	9.44911
	Community participation	7	66.67	100.00	88.0952	13.48623
	Intelligence & Vision	7	50.00	100.00	79.7619	15.85316
	Regulation & Oversight	7	87.50	100.00	96.4286	6.09938
	Transparency	7	66.67	95.83	86.9048	9.75053
	**Total Score**	**7**	**73.44**	**98.44**	**88.1696**	**8.50365**

#### Governance score stratified by residence

The overall mean score by residence was similar for peri urban and hospital and slightly higher for rural (84%). The overall score ranged from 51 to 98% in Peri urban and 64 to 98% in rural residence. For hospital based health facility scores ranged between 72% and 94%.

**Figure 2:**
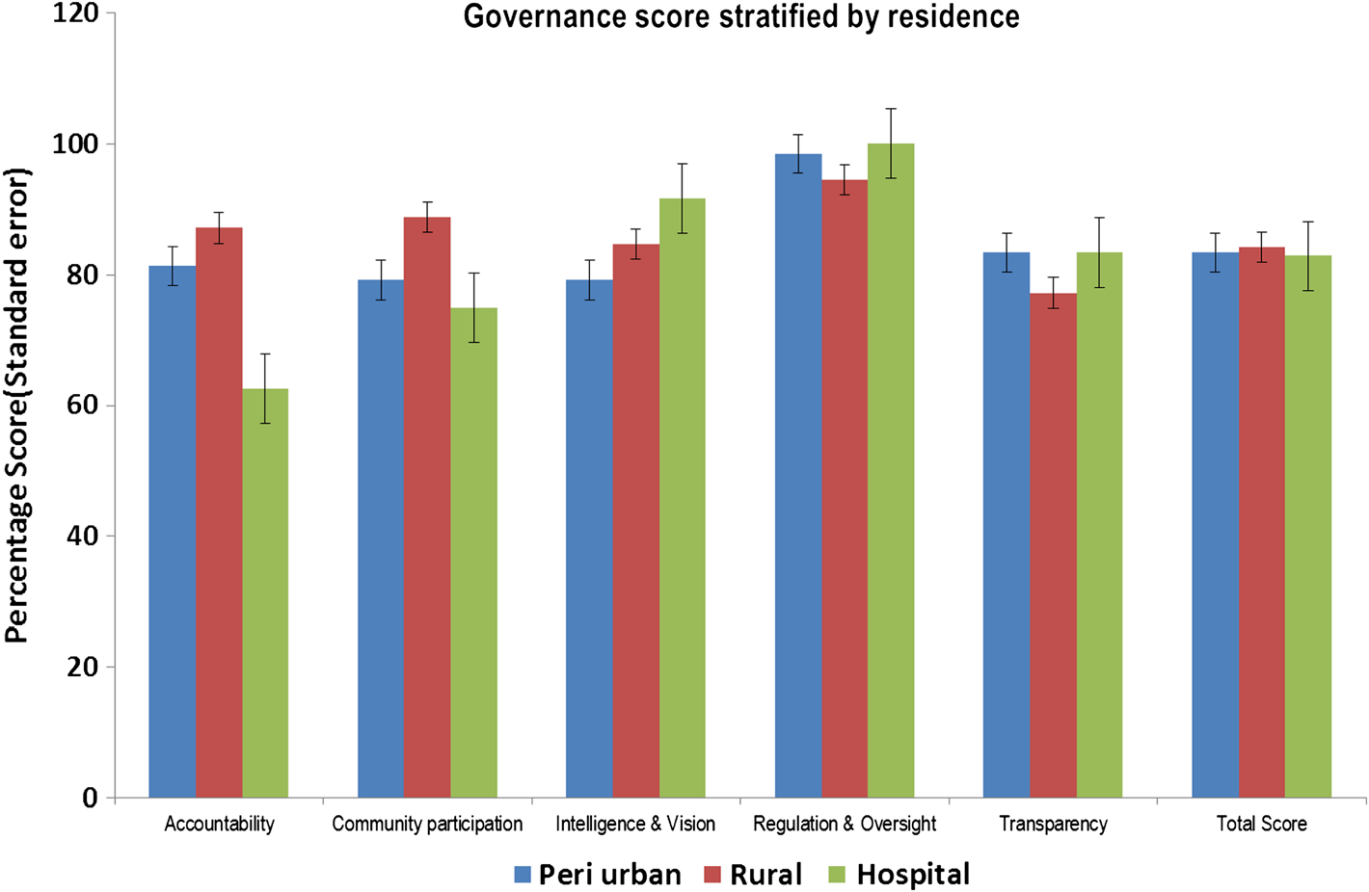
Governance scores stratified by residence.

There was variation in the score by different domains with accountability showing the highest variation. The lowest accountability score were noted in the hospital (63%) and highest in the rural health facilities (87%). Community participation was highest in the rural (89%) and lowest in the hospitals (75%). Transparency was however lower in the rural health centres when compared to peri urban and hospital based health facilities (Table 4 and Figure 2).

**Table 4:** Governance score stratified by residence

**Residence**	**Domain**	**N**	**Mini**	**Max**	**Mean**	**Std.**
**Peri urban**	Accountability	8	50.00	100.00	81.2500	18.89822
	Community participation	8	50.00	100.00	79.1667	21.82179
	Intelligence & Vision	8	50.00	100.00	79.1667	19.92048
	Regulation & Oversight	8	87.50	100.00	98.4375	4.41942
	Transparency	8	37.50	95.83	83.3333	19.28792
	**Total Score**	**8**	**51.56**	**98.44**	**83.3984**	**14.53659**
**Rural**	Accountability	32	25.00	100.00	87.1094	19.43879
	Community participation	32	50.00	100.00	88.8021	12.45231
	Intelligence & Vision	32	41.67	100.00	84.6354	15.99582
	Regulation & Oversight	32	62.50	100.00	94.5313	10.00882
	Transparency	32	25.00	100.00	77.2135	16.99885
	**Total Score**	**32**	**64.06**	**98.44**	**84.1797**	**9.21187**
**Hospital**	Accountability	2	25.00	100.00	62.5000	53.03301
	Community participation	2	50.00	100.00	75.0000	35.35534
	Intelligence & Vision	2	83.33	100.00	91.6667	11.78511
	Regulation & Oversight	2	100.00	100.00	100.0000	.00000
	Transparency	2	75.00	91.67	83.3333	11.78511
	**Total Score**	**2**	**71.88**	**93.75**	**82.8125**	**15.46796**

#### Qualitative results

Community participation and accountability were some elements of governance that showed poor performance across the study district either in isolation or in combination. We further explored these concepts using qualitative methods to establish what communities and health workers think about these elements of governance.

#### Community participation in health services

Community participation was generally low and was assumed rather than seen in practice. Most health workers interviewed said that communities participated actively in running of the health facility but at the same time acknowledged that this was mainly through community representatives who were not always active. The said community participation was inconsistent and was mainly around national campaign days such as child health week. Community participation was better in rural health facilities because of the existence of traditional structures which made it easy to organize community members. This was not the case in peri urban areas were communities were more heterogeneous and hence difficulty to organize. During focus group discussions, it was noted that there were gender differences in community participation. Male community members were more likely to participate in health facility initiatives and were well informed about services available at the health facilities and took part in the activities of the health facility. In contrast, most female participants were not aware of the activities that were going on at the clinic. However, when asked about who owned the health services at health facility, most respondents including women said that the health services were owned by the community despite their low participation.

#### Accountability for the resources

We explored the extent to which the communities or their representatives held health workers accountable for resources especially drugs and medical supplies.

Generally, it was noted that community members lacked capacity to hold health workers accountable for drugs and medical supplies. Most community members including members of Neighbourhood Health Committees (NHC) assumed that the nurses and clinical officers accounted for the drugs and did not actively ensure that this was done and appeared quite ignorant of the process of accounting for the available drugs and medicines.

## Discussion

In this baseline study, we validated a simple tool for measuring governance at health facility level. This is the first study to attempt to measure health system governance practices in Zambia with a focus on rural health centres. Most studies have focused on measuring health system governance at national, regional or district level and the questions used are usually not applicable at lower level of health system. In this study we did not attempt to measure global health governance but rather narrowed the concept of governance to the Zambian health care system and how governance can be measured at the lowest unit of health service delivery. The basis for this work was the online survey by the health system 20/20 where questions were adapted and pre-tested so that they could be applicable at the lowest level of health care in Zambia [4,9]. The results have shown that it is feasible to measure governance practices at health facility level and that the adapted tool is fairly reliable for this purpose yielding a 95% one-sided confidence interval for Cronbach’s alpha between 0.69 and 0.74 for the 16 items.

It must be mentioned from the outset that the mean scores for each item were generally higher suggesting a tendency to give higher scores by our respondents which is a common weakness with subjective evaluation [16]. Unlike the study by the 20/20 team where the respondents were not under any pressure to give higher scores, our respondents could not avoid the feeling of being evaluated by the study team despite our assurance. This bias was evident when comparing the responses to the governance questions with other observations findings on data collection and use. Generally there was little evidence on the collection and use of data yet the governance scores were still high in the items asking about collection and use of data. Despite this observation, some items had clearly low scores especially those items relating to the correction of those who do not adhere to protocols or code of practice and the availability of guidelines and protocols for child and adult health services.

Factor analysis yielded five principle components from the 16 items. One thing to note was that most of the items (6) clustered around the transparency latent factor or domain. Other latent factors had 2 or 3 items loading. The other factors identified were; community participation, intelligence& vision, accountability and regulation & oversight. These were in line with most of the governance domain suggested by Siddiqi et al., with a few elements on ethics and responsiveness not being captured by the 16 items [6]. This shows that the 16 items generally capture most of the components of governance and could be useful in comparing health facilities and tracking changes overtime. One advantage with the tool is that it is easy and quick to administer especially in busy health facilities were the managers might not have time to attend long qualitative interviews. The strength of the methodology was that rather than one person deciding the score the whole health facility team participated and came up with a consensus score for each item.

We compared baseline governance scores across the study districts and residence using the same tool. The results showed that overall score masked the clear variation across the governance domain. When domains specific comparison were made the differences between the districts and residence were very clear. Chongwe district performed poorly in overall governance score and across the five domains of governance when compared to Kafue or Luangwa districts. Most districts had poor scores in the transparency domain. Suggesting that most of the health facilities activities are not scrutinized by stakeholders or community. Our qualitative results supported these findings, as there was generally low community participation in health service delivery. We also observed that in most places, community members or their representatives were unable to hold health workers accountable for resources at the health services. Most of them were ignorant of how health workers accounted for drugs and medicines and simply trusted that it was being done well.

Residential variations were noted in the accountability and community participation which were better in rural areas and worst in hospital based health facilities. However transparency scores were lower in rural health facilities when compared to peri urban or hospital based health facilities.

The variation in the scores domain scores emphasizes the need to perform sub group analyses rather than simply relaying on overall scores which have been shown to mask the actual weakness in specific districts and residence in each domain.

The study had limitations; the overall mean scores were generally higher than anticipated. This could be attributed to the fact that this was a self administered tool and respondent were feeling pressured to give higher scores to avoid being rated low. Though the responses were based on consensus, the influence of managers on the overall response could not be eliminated though efforts were made to ensure that all the views of the respondents were considered when coming up with the final score. We also note that the results were based on 42 health facilities and therefore the results should be interpreted with caution. It is advisable to repeat the study with a larger sample size.

Despite these limitations, this tool could be useful in monitoring health system strengthening interventions targeting governance at health facility level in low income settings.

## Conclusion

The study successfully validated and applied the new tool for evaluating health system governance at health facility level. The results have shown that it is feasible to measure governance practices at health facility level and that the adapted tool is fairly reliable for this purpose with the 95% one-sided confidence interval for Cronbach’s alpha laying between 0.69 and 0.74 for the 16 items. Caution should be taken when interpreting overall scores as they tended to mask domain specific variations.

## Competing interests

The authors declare that they have no competing interests.

## Authors’ contributions

WM: Conceived the idea of applying systems thinking in evaluating the Intervention and drafted the paper. MTM: Contributed to writing of the paper and provided materials for the evaluation design. NS: Provided critical analysis and contributed to the writing of the paper. DB: Was involved in the draft of the paper and provided critical analysis of the scientific content. HA: Conceived the project and reviewed the draft and final manuscript. All authors read and approved the final manuscript.

## Pre-publication history

The pre-publication history for this paper can be accessed here:

http://www.biomedcentral.com/1472-698X/13/34/prepub
